# Percutaneous laser ablation: a new contribution to unresectable high-risk metastatic retroperitoneal lesions?

**DOI:** 10.18632/oncotarget.13897

**Published:** 2016-12-10

**Authors:** Tian’an Jiang, Zhuang Deng, Guo Tian, Fen Chen, Haiwei Bao, Ju Li, Weilin Wang

**Affiliations:** ^1^ Department of Ultrasound Medicine, the First Affiliated Hospital, Zhejiang University School of Medicine, Hangzhou, China; ^2^ Department of Hepatobiliary and Pancreatic Surgery, the First Affiliated Hospital, Zhejiang University School of Medicine, Hangzhou, China; ^3^ State Key Laboratory for Diagnosis and Treatment of Infectious Diseases, The First Affiliated Hospital, Zhejiang University School of Medicine, Hangzhou, China; ^4^ Collaborative Innovation Center for Diagnosis and Treatment of Infectious Diseases, The First Affiliated Hospital, Zhejiang University School of Medicine, Hangzhou, China

**Keywords:** laser ablation, ablation, retroperitoneal tumor, lymph nodes, oncology

## Abstract

**BACKGROUND & AIMS:**

Metastasis in retroperitoneal lymph nodes is one of the signs of advanced stage or terminal stage of malignancy. We performed a trial to assess the safety and efficacy of ultrasonography (US)-guided local neodymium-doped yttrium aluminum garnet (Nd:YAG) laser ablation for metastatic lymph nodes in the retroperitoneal region.

**METHODS:**

We evaluated 4 cases of retroperitoneal metastatic lymph nodes treated using US-guided Nd:YAG laser ablation. Additionally, we reviewed the PubMed database for articles on thermal ablation of retroperitoneal lesions until March 2016, without language limitations.

**RESULTS:**

In our study, all lesions were nearly completely ablated with mild discomfort, including pain and fever at the 3-month follow-up. In the literature review, a total of 398 patients with 491 retroperitoneal tumors were identified, and complications after the procedure included enterovesical fistula, fecal incontinence, and hematoma.

**CONCLUSIONS:**

Percutaneous laser ablation could be a theoretically promising approach for retroperitoneal metastatic lesions.ClinicalTrials.gov number: NCT02822053.

## INTRODUCTION

Local ablation of tumors has been shown to have a good curative effect [1–6]. The common ablation therapies for abdominal metastatic tumors include radiofrequency ablation (RFA) [7, 8], microwave ablation (MWA) [9], and ethanol injection (EI) [10], cryoablation [11], irreversible electroporation (IRE) [12] and high-intensity focused ultrasound (HIFU) [13]. Nevertheless, metastatic retroperitoneal lesions are a rare group of neoplasms with usual anatomical complexities, which raise challenges for radical resection. If tumors are located near great vessels, the heat effect is impaired. Additionally, retroperitoneal deep tumors, which have important structures, such as the gastrointestinal tract, in front and large blood vessels behind, can cause serious complications after injury, including death. Moreover, unintentional injury of the great vessels might result in fatal hemorrhage. Neodymium-doped yttrium aluminum garnet (Nd:YAG) laser ablation allows for accurate thermal field control, and it uses a 21-G fine needle and can penetrate the gastrointestinal tract. The adventages of the small needle and precise thermal energy distribution theoretically could minimize the risk of off-target burning and decrease problems in treating lymph nodes and particularly in difficult anatomical location. Previous studies have focused on laser ablation in the prostate [14, 15], thyroid [16–19] and liver [20, 21] while the present study aimed to assess the safety and efficacy of ultrasonography (US)-guided local Nd:YAG laser ablation for metastatic lymph nodes in the retroperitoneal region. Additionally, we performed a systematic review of the literature.

## RESULTS

In our study, the lymph node in 15-40 mm in size using 2 laser fibers. The total energy was between 2600 J and 3600 J. During laser energy application, ultrasound images showed a hyperechoic area around the fiber tip. It was obtained after a delay about 80-120s. Then the hyperechoic region expanded slowly forward. When the procedure finished, the whole lesion was covered with hyperechoic zone. There were no major complications detected in the patients during the laser ablation. All the pre-admission symptoms like abdominal pain, weakness have relieved. The detailed information during ablation was listed in Table 1.

**Table 1:** Details of the patients’ history, PLA procedure and follow-up

Patient ID	Sex	Age(year)	Primary cancer	Location	Frequency	Child-Pugh	Tumor size(necrosis size) (cm)		Number of fibers	Power (W)	Energy (J)	Distance (cm)				Prognosis	Complication/Outcome	CEA (ng/ml)		AFP (ng/mL)		CA19-9 (U/ml)	
							Before image	After image				Between the two needles	Anterior	Left	Right			Before	After	Before	After	Before	After
1	M	60	Gallbladder carcinoma	Lymph nodes around abdominal aorta		A	MR 2.5*2.1	CT 2.7*2.1 (2.5*1.8)	2	5	3600	NA	1.6; 1.5	NA	NA	PR	Fever/Alive	8.8	4.79	3.7	2.75	156	15.46
2	M	43	Liver cancer	Retroperitoneal lymph nodes	First	B	MR 3.9*3.2	CT 4*3.1	2	5	7300	1.3	1.9; 1.8	0.9	0.7	PR	Alive	1.4	1.7	1243.5	416	2.2	3.6
					Second		CT 4.1*3.1	MR 2.9*2.8 (2.5*2.2)	2	5	4500					CR							
3	M	60	Liver cancer	Retroperitoneal lymph nodes, close to the duodenum, pancreas, stomach, blood vessels	First	A	MR 3.5*2.8	CT 3*2.6	2	5	3000	0.5	1.0;1.0	0.7	0.6	PR	Alive	3.7	2.1	17.3	14.9	6.1	5.2
					Second	A	CT 3*2.6	MR 3.5*2.4	2	5	2600	0.5	1.4;1.6			PR	Alive						
					Third	A	Residual: MR 2.1*1.9		2	5	3800					PR	Alive						
					Fourth	A	Residual: CEUS: 1.05*1.02	MR 2.8*2.3 (2.5*2)	1	5	1300					CR	Alive						
4	M	60	Liver cancer	Beside the inferior vena cava		A	MR 3.9*2.1	MR 4.2*2.4(3.8*1.6)	2	5	13600	0.5				CR	Alive	2.3	1.9	3.5	2.5	17.3	3.4

M: Man; CT: Computed Tomography; MR: Magnetic Resonance; PTA: Percutaneous laser ablation;; PR: Partial response;; CR: complete response;; CEA: Carcinoembryonic Antigen;; AFP: Alpha Fetoprotein;; CA19-9: Carbohydrate Antigen 19-9.

**Figure 1:**
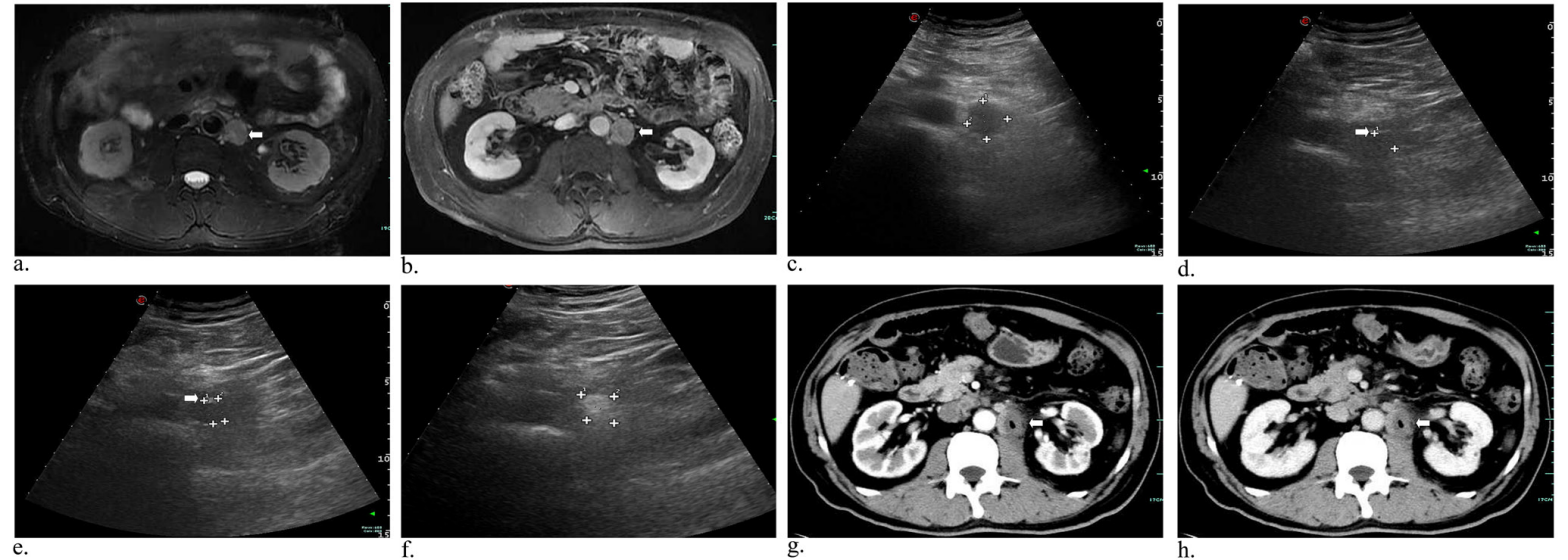
Metastasis of lymph nodes in a 60-year-old man who had undergone gallbladder cancer resection MR image of T2-weighted and substance phase showed enlarged, round lymphoma around retroperitoneal abdominal aorta a.,b. (arrowhead). Preoperative US image revealed a hypoechoic mass 2.52*2.26 cm c.. Intraoperative sonogram showed the one d. (arrowhead) and two needles e. (arrowhead) inserting into the tumor, suggesting the distances (cm) of needle tip to tumor margin (anterior: 1.61; 1.54). Afterwards, the mass had local enhancement under ultrasound scanning f., and increased unenhanced low-density areas in arterial g. (arrowhead) and substance phase h. (arrowhead) on contrast-enhanced CT scan.

**Figure 2:**
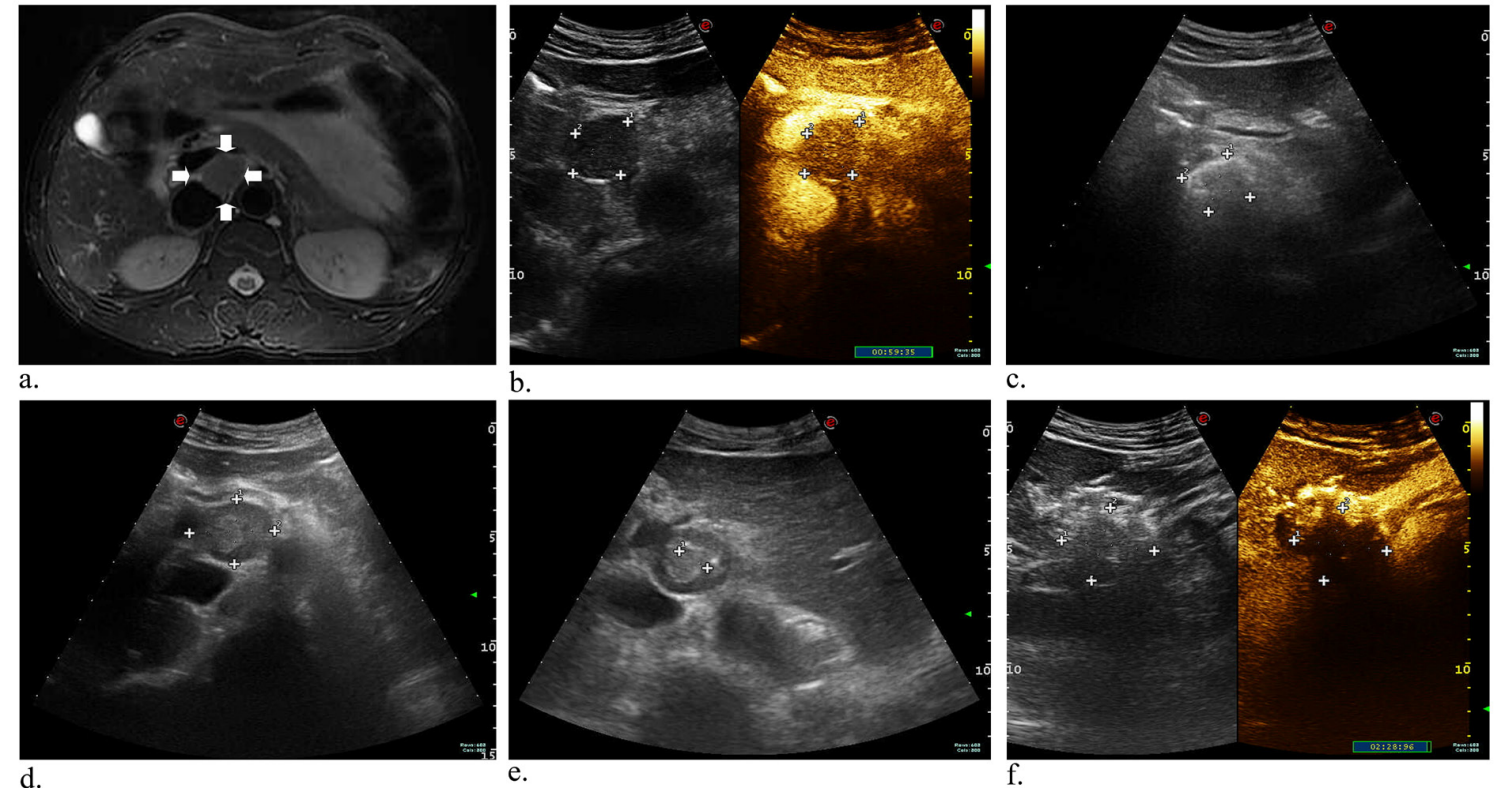
A 43-year-old man with metastatic lymph nodes originating from liver cancer Axial contrast-enhanced MR image was obtained at the abdominal setting. An oval, mildly high signal intensity is present close to aorta abdominalis a. (arrowhead). Preoperative CEUS images showed the lesion with rapid wash-in and wash-out in arterial b.. Axial gray-scale US image indicated intraoperative ethanol ablation c.. Before initial laser ablation, the mass is shown in the retroperitoneum under US guidance d.. Two laser fibers parallelly ablated the tumor under the guidance of US, which appraised the distances (cm) of needle tip to mass boundary e. (anterior: 1.9; 1.8; left: 0.9; right: 0.7), and subsequent immediate CEUS image showing a large and central filling defect f..

**Figure 3:**
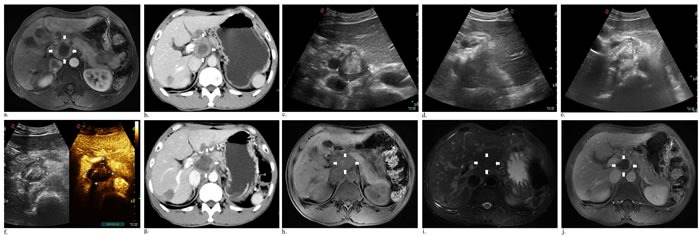
Axial abdominal MR image performed 13 days after initial ablation revealed the peripheral remanent tumor **a.** (white arrows), 6-week follow-up CT scan of the venous phase measuring 4*3.1 cm with area of central necrosis 2.3*1.8 cm **b.** (white arrows). Before the second laser ablation, a central well-defined hyperecho surrounding unenhanced hypoechoic active areas **c.**. During ablation, immediate US scan showed the left part of tumor obvious enhancement **d.**, and the next day a finding that most response appeared **e., f.**. At a follow-up visit 11 days, a contrast-enhanced CT venous phase image revealed the enlarged areas of tumor necrosis g. (white arrows). Then two months later, there was reduced mass necrosis of 2.43*1.7 cm at T1 h. (white arrows), T2 i. (white arrows) and substance phase j. (white arrows) of MR image.

**Figure 4:**
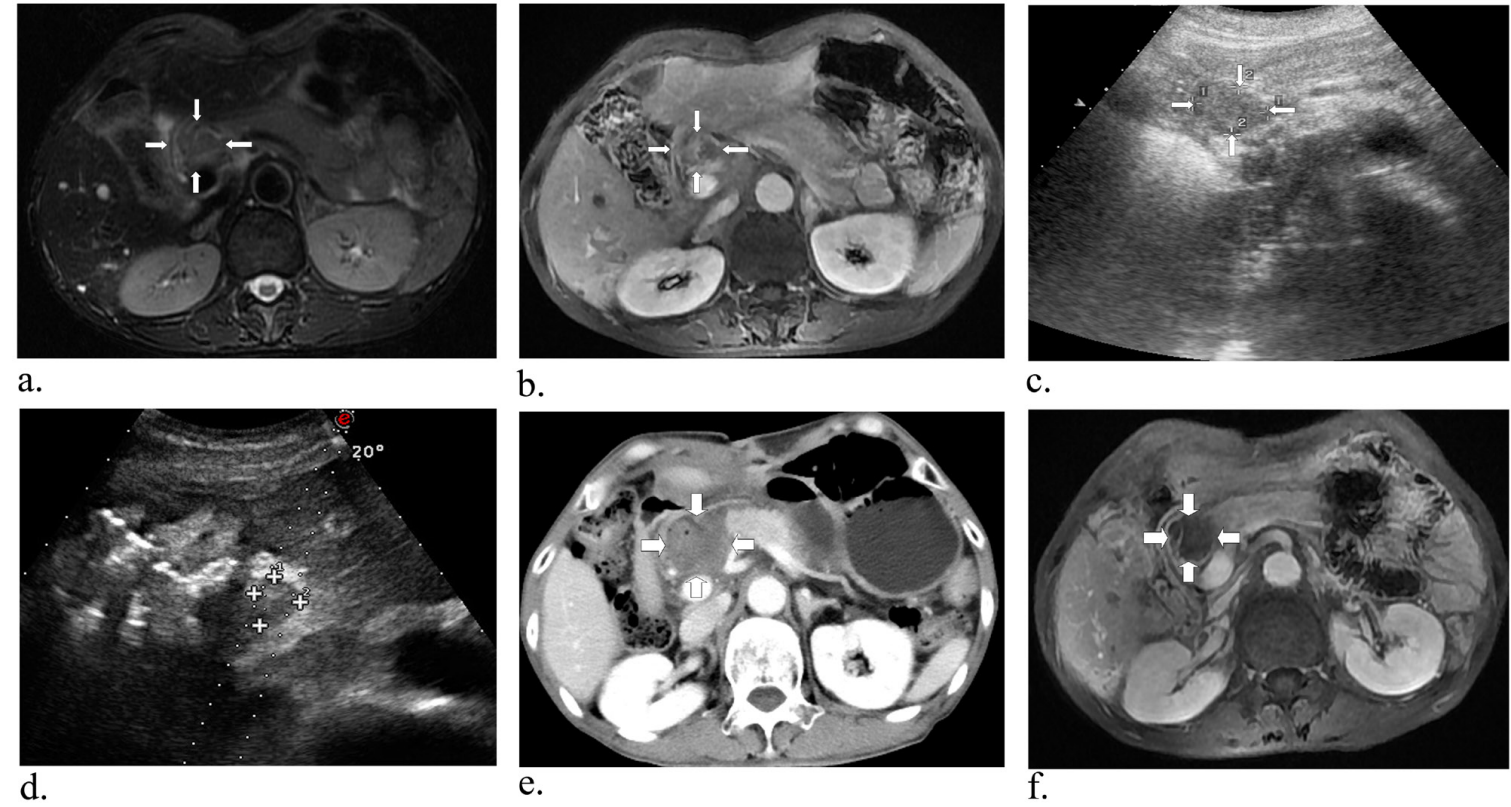
A 60-year-old man with metastatic lymph nodes close to the duodenum, pancreas, stomach and blood vessels Preoperative T2-weighted **a.** (white arrows), substance phase MR scan indicated a tumor close to the hepatic portal vein, pancreas and stomach **b.** (white arrows). Axial US image of the retroperitoneal region showed the mild hyperechoic area **c.** (white arrows). After fourth ablation, US image showed the lesion had complete response **d.** (white arrows). Substance phase of CT obtained 3 days after US-guided PLA revealed no signs of malignancy **e.** (white arrows), and then one month later, substance phase of MR image has low signal intensity **f.** showing complete necrosis of the tumour (white arrows).

**Figure 5:**
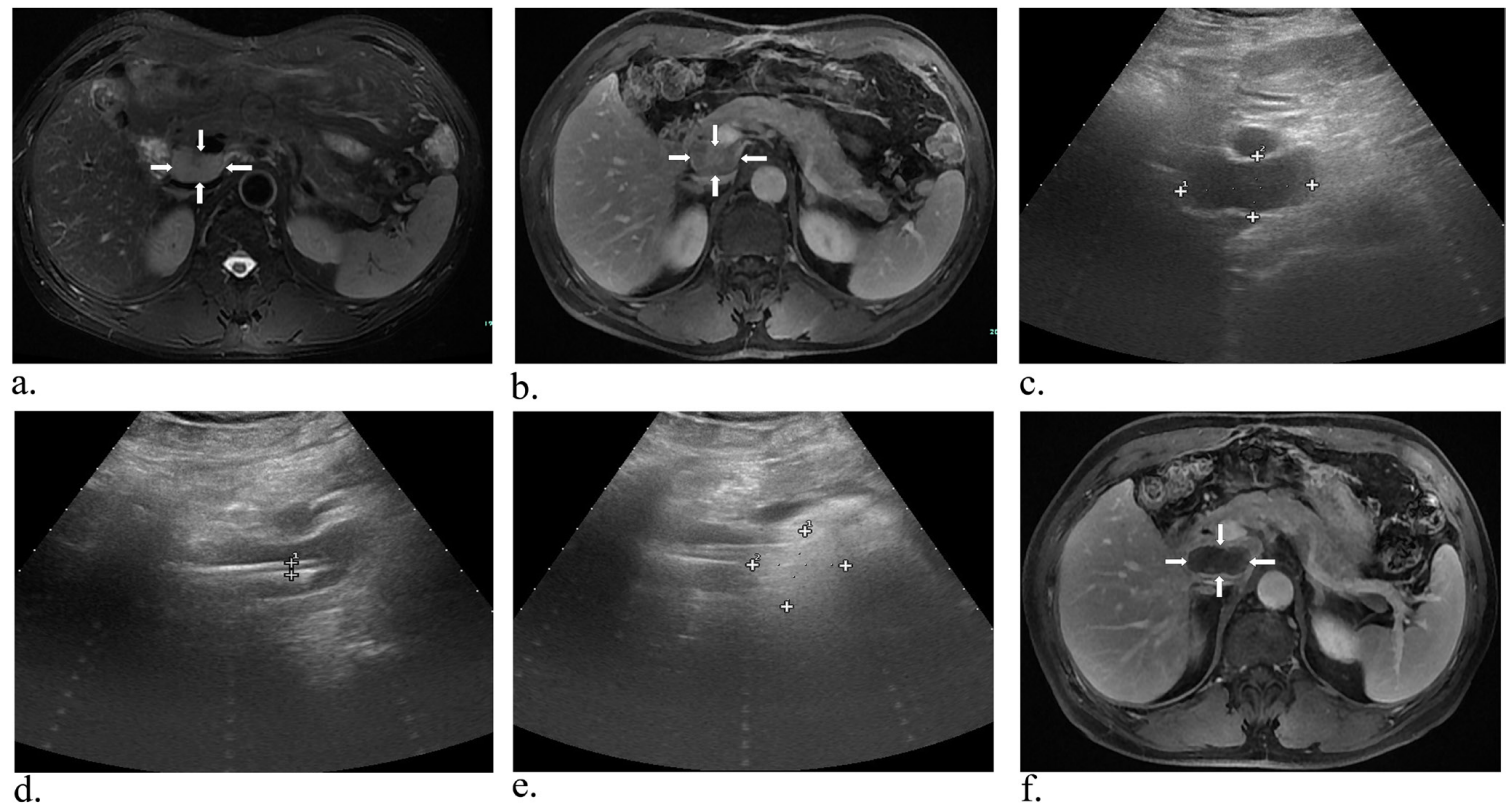
A 60-year-old man with metastatic lymph nodes beside the inferior vena cava Preoperative MR image in T2-weighted **a.** (white arrows) and substance phase **b.** (white arrows) suggested a mass of 3.9*2.1 cm in size close to the vena cava. Then with the guidance of US, two laser fibers parallelly were insered into and ablated the tumor **c.-e.** (white arrows). One month later, MRI scan indicated the lesion was complete necrosis **f.** (white arrows).

Case 1 had only single ablation with US guidance using two laser fibers due to gallbladder carcinoma metastatic retroperitoneal lymphoma, and he had decrease in CEA and CA19-9 levels while AFP level was in normal. The contrast-enhanced CT obtained 3 months after ablation showed mostly response (Figure 1). During the follow-up period, case 1 had fever for two days and went back to normal without drug intervention. After the initial laser ablation in case 2, CEA and CA19-9 levels were also declined. Two-week follow-up arterial phase of enhanced MRI revealed necrosis area of 3.2*2.3 cm, but 6 weeks later substance phase of contrast-enhanced CT scan showed the increased peripheral tumor 4*3.1 cm in size and decreased necrosis area 2.3*1.8 cm (Figure 2). Subsequently another ablation was performed and three months later MR scan indicated that the size of peripheral and necrosis area successfully reduced to 2.9*2.8 cm and 2.5*2.2 cm, respectively (Figure 3). In this study, case 3 repeatedly adopted four US-guided laser ablations in two months due to the residual lesions. CEA, AFP and CA19-9 levels were all decreased but postoperative AFP concentration still beyond reference value. Three-month follow-up MRI or CT scan showed the lymph nodes were considered as complete response for decreasing enhancement about 80% (Figure 4). In addtion, case 4 underwent one laser ablation. His tumor markers were all in normal for pre-and post-ablation. One month later, MRI indicated the lesion was complete necrosis (Figure 5).

In addition, we carefully conducted a systematic review using keywords of retroperitoneal, tumor, ablation, cryoablation, electroporation and high intensity focused ultrasound by searching from PubMed, Scoups and Web of science ahead of October 2016 without language limitations (see Appendix 1). The summary of 398 cases with 491 retroperitoneal lesions from 18 studies was presented in Table 2 [7, 8, 12, 13, 22–35]. The mean age of these studies was above 36, and 55.3% were males.

**Table 2:** Summary of 398 cases with 491 retroperitoneal tumors after ablation in 18 published literatures

Author	Year	Country	Characteristics of patients	Treatment method	Patients(No. of tumors)	Tumor size(cm)	Male/female	Mean age(range)	Follow-up interval (months)	Prognosis	Complication
Gill IS et al.	2000	America	Small renal masses	Retroperitoneal laparoscopic cryoablation	32(34)	2.3	NA	65.4 (35–93)	16.2	No evidence of local or port-site recurrence	1 perirenal hematoma;1 herpes esophagitis
Machi J et al.	2003	America	7 patients with unresectable recurrent retroperitoneal or pelvic tumors from colorectal (n=4), renal (n=2), and prostate (n=1)	US-guided RFA	7(11)	5.05 (2.9-10)	5/2	73 (62-83)	24	Local recurrencerate: 16.7%	1 enterovesical fistula;1 skin burn;1 fecal incontinence
Lee DI et al.	2003	America	Small renal masses	Retroperitoneal laparoscopic cryoablation	20(20)	2.6±0.8	11/9	67.9±13.4	14.2	1 remained unchanged	5 elevated amylase;and lipase levels;1 electrocardiogram changes;1 atrialfibrillation;1 pancreatic injury
Kariya S et al.	2005	Japan	1 metastatic left adrenal tumor from primary lung cancer;1 left renal cell carcinoma	Percutaneous CT-guided RFA with percutaneous CO2 injection	2(2)	5.5*3.8 ;3	2	67 (62-72)	1 week	Completely response	Pain
Keil S et al.	2008	Germany	1 retroperitoneal liposarcoma	Percutaneous CT-guided RFA	1(1)	2.3*2.2*3.6	1	65	27	Completely response	NA
Patel MN et al.	2008	America	Right posterior renal hilar mass	Robot-assisted cryoablation	1	3.6	1	74	4	No evidence of tumor recurrence	No
Arellano RS et al.	2010	America	3 ovarian carcinomas; 5 endometrial carcinomas	Percutaneous CT-guided RFA	8(8)	2.1 (1.0-3.7)	NA	69.1 (59-77)	23.5	5 completely response and 2 of these five died of metastatic disease at 9 and 13 months; 2 failed	NA
Orgera G et al.	2010	Italy	Hilar hepatic node from breast cancer metastasis	HIFU	1(1)	3	0/1	60	8	Completely response	NA
Wan ZH et al.	2011	China	Primary retroperitoneal sarcoma	HIFU+Surgery	47(47)	NA	26/21	56 (28-76)	60	Complete removal:74.5%;1-year recurrence rate:40.4%;5-year OS:68.1%	27 pain
Gao F et al.	2012	China	retroperitoneal metastatic lymph nodes from hepatocellular carcinoma	a)19 Percutaneous CT-guided RFA; b)13 only RFA	a.19(19); b.13(13)	a.2.2±0.1; b.2.1±0.2	a.15/4; b.10/3	a.57.3±2.3; b.52.1±2.9	9 (3-15)	1-year OS: a)26.3%;b)7.7%;the local control rate of 3, 6, 10, and 15 months: a)78.9%, 73.3%, 41.7% and 25.0%	2 hematoma;2 pain
Zhao M et al.	2012	China	retroperitoneal schwannoma	Percutaneous CT-guided RFA	2(3)	11.5*15; 7*7; 4.8*4.4	0/2	36 (22-50)	60; 27	Completely response	Pain
Littrup PJ et al.	2013	America	75 retroperitoneal soft-tissue tumors	Percutaneous CT- and/or US-guided cryotherapy	47(75)	NA	NA	60.4 (18.4-91.7)	9 (0-82)	12 total local recurrences;average time to recurrence: 5.5 months	13
Araujo LH et al.	2013	Brazil	Metastatic leiomyosarcoma	Percutaneous CT-guided RFA	1(1)	5.1*4.7	0/1	47	18	Completely response	NA
Molina R et al.	2014	Spain	Recurrence of urothelial carcinoma of the upper urinary tract after nephroureterectomy	Percutaneous CT-guided RFA	1(1)	3.1	1	73	24	Completely response	No
Narayanan G et al.	2014	America	Primary and metastatictumors in different organs	IRE	101(129)	2.7±1.5	56/45	24–83	10.3	No evidence of tumor recurrence	2 portal vein thrombosis
Monfardini L et al.	2015	Italy	Local recurrence of renal cancer after surgery	6 percutaneous RFA; 2 laparotomic RFA	8(16)	1.65 (0.7-3.4)	7/1	59 (43-77)	11.7 (7-16)	All completely response;local progression freesurvival time:11.3 months	1 abdominal fistula
Fan W et al.	2016	China	Recurrent retroperitoneal soft tissue sarcoma	CT-guided cryoablation	72(94)	1.29±0.42	29/43	49 (25–86)	45	Median PFS: 37.0 ± 7.7 months;median OS:43.0 ± 5.9 months	19 fever;11 local pain;10 emesis;6 frostbite;1 nerve injury
Underhill CE et al.	2016	America	Locally advanced pelvic and retroperitoneal tumors	IRE	15(15)	NA	8/7	54 (23-74)	3	10 margin enhancement;4 tumor ablation;1 palliation	1 urinary retention andleg paresthesias;1 foot drop

CT: Computed Tomography; RFA: Radiofrequency ablation; CR: complete response; PFS: progression-free survival; OS: overall survival; NA: Not available.

## DISCUSSION

Metastasis in retroperitoneal lymph nodes is difficult to treat, which is one of the signs of advanced stage or terminal stage of malignancy. The metastatic lymph nodes are often multiple, surrounded by large vessels, close to celiac nerve. These features lead to difficult excision for them. The routine treatments of these patients are chemotherapy or radiotherapy. But some of the tumors are not sensitive to these therapies and many patients in this period are in poor general condition, who can't tolerate the treatment procedure. Successful local inactivation of these lymph nodes can prolonged survival in patients [7, 28]. Previous studies about thermal ablations such as percutaneous CT and/or US-guided RFA, cryotherapy of the retroperitoneal tumors showed that the complications usually occurred in postoperation [7, 8, 12, 13, 22–35]. While the diameter of the electrode used in RFA is usually 14- to 17-gauge, which could be easily bleeding. As we know the retroperitoneal lymph node is seated in deep position. In front of them, there are bowels, stomach, pancreas, or liver. If the electrode passes through these structures, severe complications will occur. Furthermore, the lymph nodes are adjacent to abdominal aorta and inferior vena cava, which are risky that can't be harmful. Thus these make the treatment difficult. Some studies reported that CT-guided interventional therapies were conducted, whcih could provide precious position of the puncture needle and the retroperitoneal tumors [8, 28]. Ablation in the prone position could avoid anterior bowel loops and vascular structures under CT guidance [36, 37]. But sometimes it could be forbidden in CT examination when the lymph node was covered by some important regions like the bowel. In this study, ultrasonography could freely guide the needle into the deep position from more angles. So it was feasible to keep the needle away from the vessels and organs. In addition, in recent years, real-time fusion imaging of US and preoperative CT or MRI images has been considered to be helpful in targeting tumors in complex and delicate anatomical area [38, 39], which could be a promising way to focus on this disease.

Laser ablation is one of the thermal therapies that used for local control of malignant tumor. In this study, we used ultrasonography to guide the inserting of Nd:YAG laser fiber and no major complications occurred. It was a theoretically good adjuvant tool because it enabled the real-time visualization of the needle and the target lesion. The color Doppler imaging allowed the clear identification of the related vessels along the route. Moreover, intraprocedural use of CEUS could immediately increase lesion conspicuity and decrease the number of incomplete treatments and re-treatments. CEUS was regarded as accurate as other contrast-enhanced imaging modalities for the evaluation of technical success after ablation, which had better cost-effectiveness compared with the standard procedure [38, 40]. It was reported the needle tract implantation metastases rate from 0% to 4.4% in RFA [41–43], which enabled coarse needle inserting and even penetrating into the bottom of the tumor, then needle ablation against bleeding and implantation metastases. But in PLA, radiologist used the most fine needle and slim optical fiber to locate the tip in the shallow tumor, when finished, retreating fiber to the sheath and the needle ablation. To date, there has been no report of needle implantation metastases in PLA. In addition, PLA use YAG laser through optical fiber transmission without complications of electric injury, vagus nerve excitement caused by current, and also available in patients with severe heart disease, arrhythmia, cardiac pacemaker.

In most studies, they were reported that the lesion close to the great vessels like hepatic vein was likely to cause residual tumor because the thermal energy was taken away by the blood flow [44, 45]. In this study, these lymph nodes were either close to the aorta, inferior vena cava or the portal vein. Tumor markers decreased as well. It showed the laser ablation is effective in the lesions of risky positions. The reason may result in that comparing with the radiofrequency and microwave, Nd:YAG laser has a shorter wavelength of 1064 nm and higher accuracy of ablation, as well as the characteristics of ultrasonic. As is known to all, the higher the ablation temperature is, the more tissue dehydration, and the stronger coagulability. Comparing with other ablations, PLA is a technology of higher central and surrounding temperature with low incidence of splitting or falling off in ablation lesion. It clinically has the output power of 1 to 7 w but 10 w to 100 w in RFA and WMA. Several studies reported that excessive energy input may trigger malignant tumor recurrence and sharp expansion [46–48]. Therefore, on the premise of effectively inactivating tumor, the lower power and energy will generate better prognosis. Furthermore, laser ablation obtained pathologically and microscopically clear and thin boundary between the ablated solidification area and the surrounding normal tissues while irregular and thick using RFA and MWA [49]. Therefore it is theoretically safer when treating the lesions close to great vessels. It meaned that the treatment was of the beneficial.

Our results should be noted in view of several limitations. First, the limited sampling size and the short-term follow-up were noticeable, which may bring results bias. Second, few studies had reported whether this technique was appropriate for metastatic lymph nodes in such patients, in particular without multi-center controlled trials. Thirdly, although we seriously conducted US-guided laser ablation for retroperitoneal lesions, some complex critical lesions under single US guidance could be still undetectable, and real-time fusion imaging of US and CT or MRI images could be applied to identify clearer depiction of retroperitoneal anatomical regions in the future.

Taken together, US-guided percutaneous laser ablation could be theoretically promising for unresectable metastatic retroperitoneal lymph nodes and hepatic portal lymph nodes, with few complications and a small and precise ablation area. However, large-scale studies on laser ablation for metastatic retroperitoneal lymph nodes are necessary to confirm our findings.

## MATERIALS AND METHODS

During the last 8 months, four patients (4 men, age range 43-60 years old, mean age 56 years old) suffering from a painful retroperitoneal lymphoma underwent US-guided laser ablation. Involved primary cancers included 1 gallbladder carcinoma (GC), 3 hepatocellular carcinomas (HCC). The targeted metastatic lymph nodes in the study were 4 in retroperitoneum (case 1: around abdominal aorta; case 2: retroperitoneum; case 3: retroperitoneum, close to the duodenum, pancreas, stomach, blood vessels; case 4: beside the inferior vena cava). This clinical trial was registered inClinicaltrials.gov ID: NCT02822053 on June 20th 2016. The procedure was approved by the ethics committee of The First Affiliated Hospital of Zhejiang University. All authors had access to the study data and have approved and reviewed the final manuscript. All of patients have history of partial hepatectomy. Furthermore, case 2 undergoing two percutaneous ethanol injections (PEI) last year, which did not control tumor growth, had a mass in size increased from 2.9*2.2 cm to 3.8*2.9 cm through MR images examination. The basic information of the three patients was listed in Table 1. Lymph nodes size (maximum diameter) ranged from 1 cm to 4.0 cm (mean 2.9 cm). Patients were symptomatic including experienced abdominal pain, weakness and weight lost. Child-Pugh is an clinically classified standard to quantitatively assess the liver reserve function in patients with cirrhosis, and it includes 5 indicators (hepatic encephalopathy, ascites, total bilirubin, serum albumin, and prothrombin time). The liver reserve function of different severity of liver damage could be assessed using A, B, C levels. Preoperative and postoperative tumor markers of Carcinoembryonic Antigen (CEA), Alpha Fetoprotein (AFP) and Carbohydrate Antigen 19-9 (CA19-9) levels were measured. In this study, all lymph nodes metastases were histologically confirmed using US-guided biopsy before laser ablation. The tumor size and location near organs and vascular structures were evaluated using computed tomography (CT) or magnetic resonance image (MRI) and US. The efficacy of local ablation could be sorted into four types of complete response (CR), partial response (PR), no change (NC) and lymph node progressive (LP) as the World Health Organization (WHO) response evaluation criteria for solid tumor [50].

At first, the radiologists used CEUS with 2.4 mL SonoVue (Bracco, Milan, Italy) to accurately target the lesions. After the correctly positioning of the tip of the needle, a plane-cut optic fiber (300 μm in diameter) along with the sheath of a 21-G needle was advanced with a 10-mm bared fiber into the lesions. Then laser ablation under ultrasonographic guidance was performed by a Nd:YAG laser-beam fiber (EchoLaser X4, ESAOTE, Italy) at a wavelength of 1064 nm. The output power of laser was 5 W and the time was approximately 5-6 minutes. Power and energy were selected based on previous experience [51]. Immediately after laser ablation, the entire area without enhancement under CEUS was defined as success. If still enhanced in the target lesion, it was regarded as residual area. Then both the fiber and the sheath were withdrawn about 2-5 mm. Supplementary energy was applied to destroy an area larger than the lesional volume. The selective number of fibers was based on the size of the lesion. In this study, all statistical analyses were performed using spss 13.0 software.

## SUPPLEMENTARY MATERIALS FIGURES AND TABLES




