# Supplementary material for: Percutaneous laser ablation: a new contribution to unresectable high-risk metastatic retroperitoneal lesions?
Source: Oncotarget. 2016 Dec 10;8(2):2413–22. doi: 10.18632/oncotarget.13897 (PMC5356811; doi:10.18632/oncotarget.13897)
Supplement: Supplementary file 1 [file oncotarget-08-2413-s001.pdf]

Percutaneous laser ablation: a new contribution to unresectable high-risk metastatic retroperitoneal lesions?

Background and rationale

Metastasis in retroperitoneal lymph nodes is one of the signs of advanced stage or terminal stage of malignancy. The common ablation therapies for abdominal metastatic tumors include radiofrequency ablation (RFA), microwave ablation (MWA), and ethanol injection (EI), cryoablation, irreversible electroporation (IRE) and high-intensity focused ultrasound (HIFU). Nevertheless, metastatic retroperitoneal lesions are a rare group of neoplasms with usual anatomical complexities, which raise challenges for radical resection. If tumors are located near great vessels, the heat effect is impaired. Additionally, retroperitoneal deep tumors, which have important structures, such as the gastrointestinal tract, in front and large blood vessels behind, can cause serious complications after injury, including death. Moreover, unintentional injury of the great vessels might result in fatal hemorrhage. Neodymium-doped yttrium aluminum garnet (Nd:YAG) laser ablation allows for accurate thermal field control, and it uses a 21-G fine needle and can penetrate the gastrointestinal tract.

Objectives

The purpose of this study is to assess the safety and efficacy of ultrasonography (US)-guided local neodymium-doped yttrium aluminum garnet (Nd:YAG) laser ablation for metastatic lymph nodes in the retroperitoneal region.

Study design and methodology

This was designed as an open-label, pilot study. The clinical trial was registered in Clinicaltrials.gov ID: NCT02822053 on June 20th 2016. All patients and relatives were informed about the purpose and procedures of this study and gave written informed consent. The study was approved by the Institutional Review Board at the First Affiliated Hospital of Zhejiang University School of Medicine, Hangzhou and all studies were conducted in accordance with relevant guidelines and regulations. The investigators used preoperative and postoperative US/CEUS/CT/MRI to assess lesions, and laboratory tests including the tumor markers to evaluate the general condition of patients. All patients were followed up for 1–2.5 years as scheduled. Inclusion criteria were as follows:1) unresectable high-risk metastatic retroperitoneal lesions; 2) Child-Pugh A/B; 3) PLT $\geqslant$ 50*10E9/L and PT $\leqslant$ 20s. Exclusion criteria were as follows: patients with blood coagulation dysfunction, ChildPugh $\geqslant$3, severe cardiopulmonary disease, intolerant anesthesia and upper gastrointestinal bleeding in shock.

Statistical analyses

In this study, all statistical analyses were performed using spss 13.0 software.

Organization of the clinical trial

The First Affiliated Hospital of Zhejiang University School of Medicine, Hangzhou.
