# Supplementary material for: Percutaneous laser ablation: a new contribution to unresectable high-risk metastatic retroperitoneal lesions?
Source: Oncotarget. 2016 Dec 10;8(2):2413–22. doi: 10.18632/oncotarget.13897 (PMC5356811; doi:10.18632/oncotarget.13897)
Supplement: Supplementary file 2 [file oncotarget-08-2413-s002.pdf]

Appendix 1

Search strategy:

Pubmed (1950-present)

1. ("posterior peritoneum" OR "retroperitoneal")
2. (neoplas* OR cancer OR tumor OR tumour OR carcinoma OR oncolog*)
3. (ablation OR cryoablation OR cryotherpay OR electroporation OR HIFU OR (High Intensity Focused Ultrasound)
4. 1 AND 2 AND 3
5. "Retroperitoneal Neoplasms"[Mesh]
6. "Retroperitoneal Space"[Mesh]
7. "Retroperitoneal liposarcoma"[Mesh]
8. 5 OR 6 OR 7
9. "Ablation Techniques"[Mesh]
10. "High-Intensity Focused Ultrasound Ablation"[Mesh]
11. "Electroporation"[Mesh]
12. "Electrochemotherapy"[Mesh]
13. 9 OR 10 OR 11 OR 12
14. 8 AND 13
15. 4 OR 14

Scoups

1. TITLE-ABS-KEY (“posterior peritoneum”)
2. TITLE-ABS-KEY (“retroperitoneal”)
3. 1 OR 2
4. TITLE-ABS-KEY ("neoplas*")
5. TITLE-ABS-KEY ("cancer")
6. TITLE-ABS-KEY ("tumor")
7. TITLE-ABS-KEY ("tumour")
8. TITLE-ABS-KEY ("carcinoma")
9. TITLE-ABS-KEY ("oncolog*")
10. 4 OR 5 OR 6 OR 7 OR 8 OR 9
11. TITLE-ABS-KEY ("ablation")
12. TITLE-ABS-KEY ("cryoablation")
13. TITLE-ABS-KEY ("cryotherpay")
14. TITLE-ABS-KEY ("electroporation")
15. TITLE-ABS-KEY ("HIFU")
16. TITLE-ABS-KEY ("High Intensity Focused Ultrasound")
17. 11 OR 12 OR 13 OR 14 OR 15 OR 16
18. 3 AND 10 AND 17

Web of science

1. TS=(Retroperitoneal Neoplasms)
2. TS=(Retroperitoneal Space)
3. TS=(Retroperitoneal liposarcoma)
4. 1 OR 2 OR 3
5. TS=(Ablation Techniques)
6. TS=(High-Intensity Focused Ultrasound Ablation)
7. TS=(Electroporation)
8. TS=(Electrochemotherapy)
9. 5 OR 6 OR 7 OR 8
10. TI=(posterior peritoneum)
11. TI=(retroperitoneal)
12. 10 OR 11
13. TI=(neoplas*)
14. TI=(cancer)
15. TI=(tumor)
16. TI=(tumour)
17. TI=(carcinoma)
18. TI=(oncolog*)
19. 13 OR 14 OR 15 OR 16 OR 17 OR 18
20. TI=(ablation)
21. TI=(cryoablation)
22. TI=(cryotherpay)
23. TI=(electroporation)
24. TI=(HIFU)
25. TI=(High Intensity Focused Ultrasound)
26. 20 OR 21 OR 22 OR 23 OR 24 OR 25
27. 4 AND 9 AND 12 AND 19 AND 26

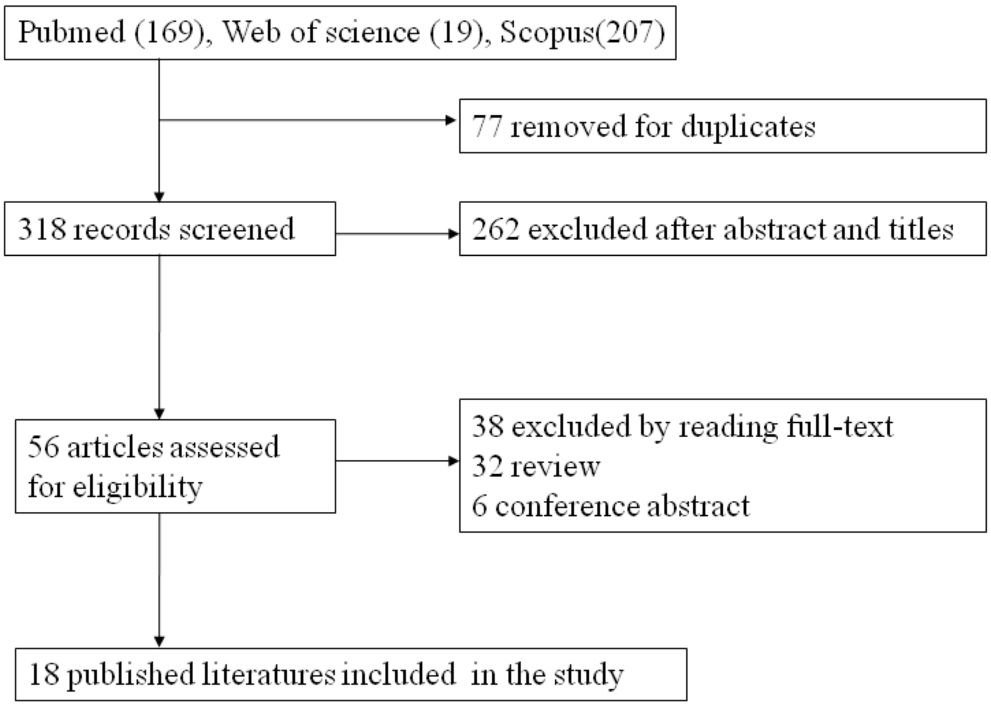

Figure S1: Flow chart of study selection.
